# Tea consumption and risk of all-cause, cardiovascular disease, and cancer mortality: a meta-analysis of thirty-eight prospective cohort data sets

**DOI:** 10.4178/epih.e2024056

**Published:** 2024-06-21

**Authors:** Youngyo Kim, Youjin Je

**Affiliations:** 1 Department of Food and Nutrition/Institute of Agriculture and Life Science, Gyeongsang National University, Jinju, Korea; 2 Department of Food and Nutrition, Kyung Hee University, Seoul, Korea

**Keywords:** Tea, Mortality, Cardiovascular diseases, Cancer, Meta-analysis

## Abstract

**OBJECTIVES:**

Tea consumption has been considered beneficial to human health because tea contains phytochemicals such as polyphenols and theaflavins. We conducted a systematic review and meta-analysis on the association between tea consumption and mortality from all causes, cardiovascular disease (CVD), and cancer to provide a quantitative assessment of current evidence.

**METHODS:**

The PubMed, Web of Science, and Scopus databases were searched through April 2024 to identify eligible studies. Random effects models were used to combine study-specific effect estimates (ESs).

**RESULTS:**

A total of 38 prospective cohort data sets (from 27 papers) with 1,956,549 participants were included in this meta-analysis. The pooled ESs of the highest versus lowest categories of tea consumption were 0.90 (95% confidence interval [CI], 0.86 to 0.95) for all-cause mortality, 0.86 (95% CI, 0.79 to 0.94) for CVD mortality, and 0.90 (95% CI, 0.78 to 1.03) for cancer mortality. In the dose-response analysis, a non-linear association was observed. The greatest risk reductions were observed for the consumption of 2.0 cup/day for all-cause mortality (ES, 0.91; 95% CI, 0.88 to 0.94) and 1.5 cup/day for cancer mortality (ES, 0.92; 95% CI, 0.89 to 0.96), whereas additional consumption did not show a further reduction in the risk of death. A plateau was observed for CVD mortality at moderate consumption levels (1.5-3.0 cup/day), but a sustained reduction in mortality risk was observed at higher intake levels.

**CONCLUSIONS:**

Moderate tea consumption (e.g., 1.5-2.0 cup/day) was associated with lower all-cause, CVD, and cancer mortality compared to no tea consumption. Further well-designed prospective studies are needed for a definitive conclusion.

## GRAPHICAL ABSTRACT

[Fig f8-epih-46-e2024056]

## Key Message

Tea is a commonly consumed beverage worldwide and has a significant public health impact. The association between tea consumption and risk of mortality from chronic disease remains inconsistent, and extensive cohort studies have been published recently. In this meta-analysis, including thirty-eight cohort studies, people who drank one and a half to two cups of tea daily had a lower risk of mortality from all causes, cardiovascular disease, and cancer than those who drank less tea.

## INTRODUCTION

Tea has been one of the most commonly consumed beverages worldwide for centuries [1]. Tea is a rich source of flavonoids such as catechins, theaflavins, anthocyanins, and flavonols [2]. Flavonoids are known for their potential to reduce the risk of many non-communicable diseases, including cardiovascular disease (CVD) and cancer, due to their anti-inflammatory and antioxidant properties that reduce oxidative stress [3,4]. In addition, a meta-analysis involving 386,610 participants showed that high flavonoid intake was associated with a lower risk of mortality from CVD and all causes [5].

Because tea is widely consumed globally, its effect on public health can be major even if its effect is minor on an individual level. Many observational studies have explored the associations between tea consumption and the risks of all-cause and cause-specific mortality [6-32]. Some studies found an inverse [7,17,21-24,27,28,30] or positive [9,10] association between tea consumption and the risk of mortality, whereas others did not find a significant association [6,8,11-16,18-20,25,26,29,31,32]. To date, several meta-analyses have been conducted to examine the association between tea consumption and risk of mortality, but they included studies in which all participants had a specific disease [33,34] or did not provide results for cancer mortality [34,35]. Since then, many large cohort studies have been published, analyzing data from the UK Biobank (n=498,158) and Asia Cohort Consortium (n=528,504) [27-32]. Recent large cohort studies have indicated that significant associations exist between tea consumption and mortality [27,28,30]. Given the results published to date, we speculated about the possible association between tea consumption and mortality if a meta-analysis were conducted on the general population, excluding as many studies as possible that targeted only patients.

Therefore, we performed a systematic review and meta-analysis of prospective cohort studies to provide the latest evidence on the associations between tea consumption and the risk of mortality from all causes, CVD, and cancer in the general population. Furthermore, a dose-response analysis was conducted to determine trends in mortality risk with increased tea consumption and elucidate appropriate levels of tea consumption to reduce the risk of death.

## MATERIALS AND METHODS

### Data sources and searches

A systematic literature search was performed using the PubMed, ISI Web of Science, and Elsevier Scopus databases to identify eligible studies published in English through April 2024. We used the following keywords in the search: “(tea OR beverage OR beverages) AND (mortality OR death OR fatal).” A manual search that reviewed the included articles’ reference lists was also conducted to identify additional eligible studies. This meta-analysis was registered on PROSPERO (CRD42023400484).

### Study selection

Studies were included in the current meta-analysis according to the following criteria: (1) the study had a prospective observational design; (2) the exposure of interest was tea consumption; (3) the outcome of interest was mortality from all causes, CVD, and cancer; (4) the researchers reported effect estimates (ESs; relative risks [RRs] or hazard ratios [HRs]) with the corresponding 95% confidence intervals (CIs) for each tea-intake group. If 2 different papers had been published on the same cohort data sets, the study with a more extended follow-up period or more participants was included in the meta-analysis. If 2 papers following the same cohort reported deaths from different causes, both were included in the meta-analysis [19,32]. Studies in which the participants consisted only of patients with a specific disease were excluded.

#### Data extraction and quality assessment

The relevant data were independently extracted by 2 authors (YK and YJ) according to the Preferred Reporting Items for Systematic Reviews and Meta-Analyses (PRISMA) statement [36]. The following information was collected from each paper: surname of first author, year of publication, cohort name, number of participants and cases, age, gender, geographical region or country, period of follow-up, categories of tea consumption, the ESs (RRs or HRs) and 95% CIs for each category of tea consumption, and adjusted variables.

Quality assessment of the included studies was performed using the Newcastle-Ottawa quality assessment scale [37]. We assigned a maximum score of 9 points to each study based on the following criteria: selection of participants (0-4 points), comparability of cohorts (0-2 points), and ascertainment of outcomes of interest (0-3 points). Studies with total scores of 0-3 points, 4-6 points, or 7-9 points were regarded as low, moderate, or high quality, respectively. Any discrepancies between authors in data extraction and quality assessment were resolved by reviewing and discussing the original papers.

### Statistical analysis

The natural logarithm values of each study’s ESs (RRs or HRs) were combined using a DerSimonian & Laird [38] random effects model, which considers both within-study and between-study variability. If a study provided separate ESs by the presence or absence of disease history, we used the ESs for the latter [32]. The summary ESs were displayed as forest plots. The Cochran Q test [39] was used to assess heterogeneity among studies, and the *I*^2^ statistic [40] was used to quantify inconsistency. Subgroup analyses stratified by gender, geographic region, follow-up years, and sample size were conducted to investigate the variations in ESs among the studies. To assess how much a specific study may influence inferences, we performed a senstivity analysis by omitting a single study at a time and deriving pooled ESs. Publication bias was evaluated with the Begg test [41] and the Egger regression test [42], as well as funnel plot visualizations.

To estimate the study-specific slope lines, a linear dose-response meta-analysis was conducted using the methods proposed by previous studies [43-45]. The median value of tea consumption was assigned to each category based on information presented in the original paper. When the highest category was open-ended, we regarded it as of the same magnitude as the adjacent category. Referring to previous papers, we considered 150 mL of tea consumption to be 1 cup of tea [9,33]. To investigate a potential nonlinear association between tea consumption and risk of mortality, we used restricted cubic splines with 4 knots at fixed percentiles (5th, 35th, 65th, and 95th percentiles) of aggregated tea consumption. The p-value for non-linearity was computed by testing the null hypothesis that the regression coefficient of the second spline is equal to zero [46]. A 2-tailed p-value of less than 0.05 was considered to indicate statistical significance. All statistical analyses were carried out with Stata version 17.0 (StataCorp., College Station, TX, USA).

## RESULTS

### Study characteristics

**Figure 1.**
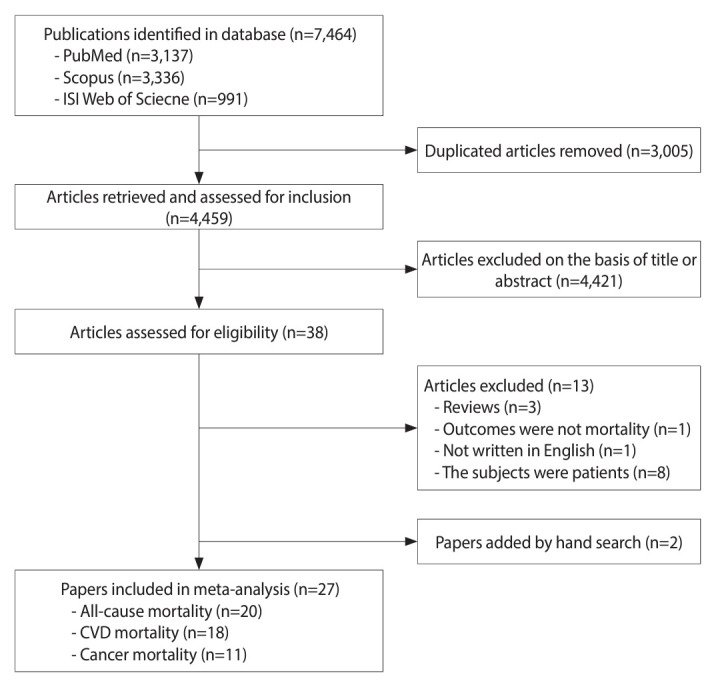
Flow chart of study selection.

A total of 38 prospective cohort data sets (27 papers) including 1,956,549 participants and 218,948 deaths from all causes, 53,234 deaths from CVD, and 56,049 deaths from cancer were eligible for this meta-analysis (Figure 1) [6-32]. Table 1 summarizes the characteristics of the included studies. Among them, 7 papers reported all estimates of mortality from all causes, CVD, and cancer [17,21-23,25,26,30], respectively. Six papers provided estimates of mortality from all causes and CVD [10,15,18,24,27,28], and 1 paper reported estimates of mortality from all causes and cancer [9]. The other papers provided mortality from either all causes [6,8,13,16,31,32], CVD [7,11,12,19,20] or cancer only [14,29]. The follow-up durations varied from 5 years to 23 years, and the mean follow-up time was 12.3 years. The studies were conducted in Asia [11,13,14,17,19,22,27,30-32], Europe [7,9,10,12,18,26,28,29], United States [6,8,15,16,20,21,23,25], and Oceania [24]. All studies were adjusted for age and smoking, and most were adjusted for alcohol consumption [7,9-11,13,15-19,21-23,25-32] and body mass index (BMI) [7-12,15-17,19-32]. The quality assessment results ranged from 7 to 9, indicating high quality (Supplementary Materrial 1).

### All-cause mortality

**Figure 2.**
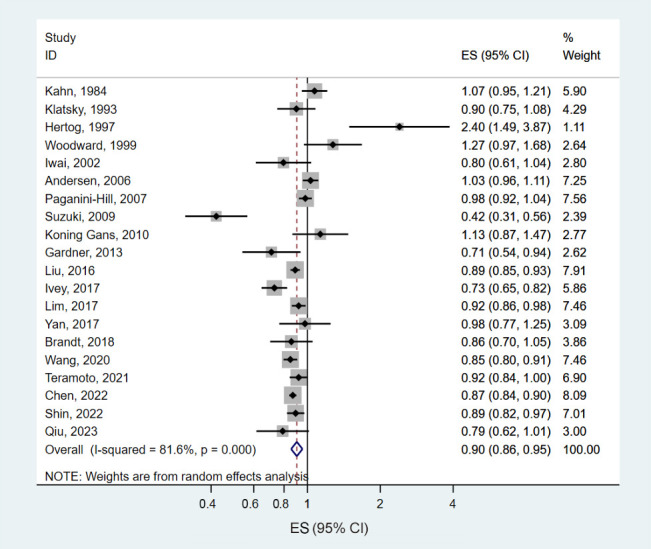
Forest plots of the prospective cohort studies of all-cause mortality for high versus low tea consumption. ES, effect estimate; CI, confidence interval.

**Figure 3.**
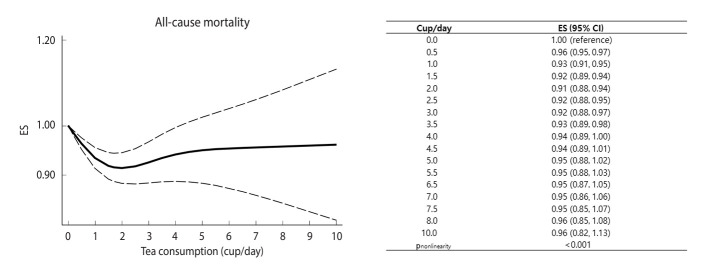
Pooled dose-response association between tea consumption and all-cause mortality. Solid lines represent effect estimates (ESs); dashed lines represent 95% confidence intervals (CIs).

Thirty-three prospective cohort data sets (20 papers) involving 1,825,815 participants and 218,948 deaths examined the association between tea consumption and all-cause mortality [6,7,9,10, 13,15-18,21-28,30-32]. The summary ES for all-cause mortality, comparing the highest and lowest levels of tea consumption, was 0.90 (95% CI, 0.86 to 0.95), with significant heterogeneity among studies (*I*^2^ = 81.6%, p<0.001) (Figure 2). The sensitivity analysis results, excluding 1 study at a time, showed that the pooled ESs ranged from 0.89 (95% CI, 0.85 to 0.94) to 0.92 (95% CI, 0.88 to 0.96) (Supplementary Material 2). The inverse association between tea consumption and the risk of all-cause mortality was slightly stronger in men (ES, 0.87; 95% CI, 0.77 to 0.98) than in women (ES, 0.90; 95% CI, 0.79 to 1.01), but the difference was not statistically significant (p for difference=0.97) (Table 2). By geographic region, the strongest inverse association between tea consumption and all-cause mortality was shown in Asia (ES, 0.84; 95% CI, 0.77 to 0.91), while an insignificant positive association was observed in Europe (ES, 1.12; 95% CI, 0.88 to 1.42; p for difference=0.05). We did not observe significant variation according to follow-up times (p for difference=0.17) or number of participants (p for difference=0.87). We found a non-linear association between tea consumption and all-cause mortality (p for non-linearity < 0.001) (Figure 3). A significant reduction in all-cause mortality was observed with up to 2.0 cup/day of tea consumption, and additional consumption did not show a further decrease in mortality.

#### CVD mortality

**Figure 4.**
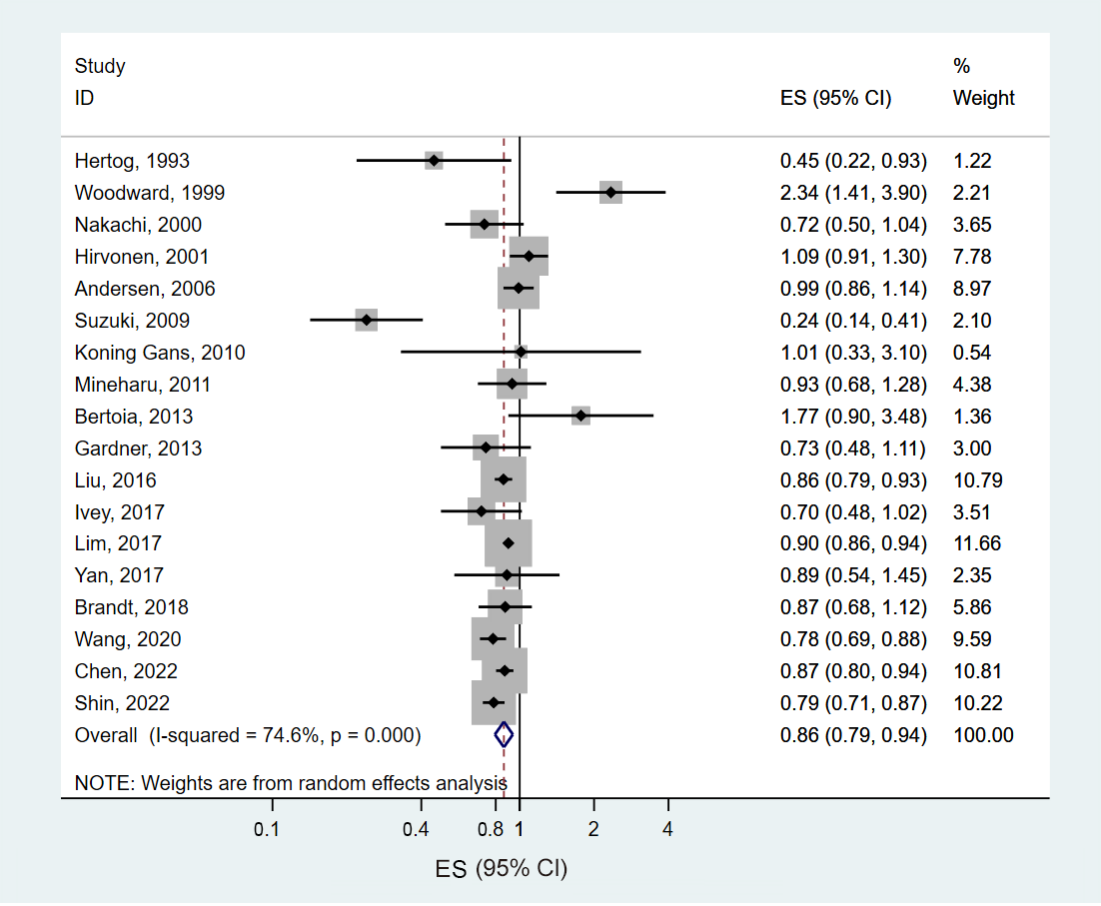
Forest plots of the prospective cohort studies of cardiovascular disease mortality for high versus low tea consumption. ES, effect estimate; CI, confidence interval.

**Figure 5.**
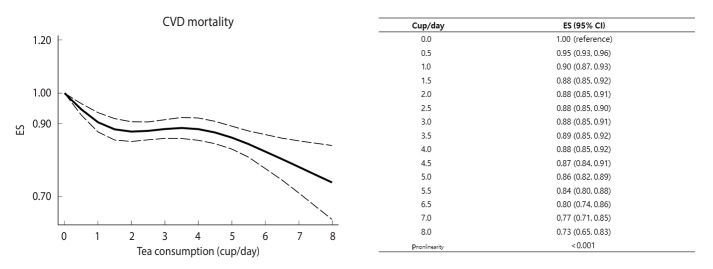
Pooled dose-response association between tea consumption and cardiovascular disease (CVD) mortality. Solid lines represent effect estimates (ESs); dashed lines represent 95% confidence intervals (CIs).

Thirty-one prospective cohort data sets (18 papers) involving 1,820,381 participants and 53,234 deaths examined the association between tea consumption and CVD mortality [7,10-12,15,17-28,30]. The summary ES for CVD mortality, comparing the highest and lowest levels of tea consumption, was 0.86 (95% CI, 0.79 to 0.94), showing significant heterogeneity among studies (*I*^2^= 74.6%, p<0.001) (Figure 4). When the pooled ES was estimated after omitting 1 study at a time, the ESs ranged from 0.85 (95% CI, 0.78 to 0.92) to 0.88 (95% CI, 0.82 to 0.95) (Supplementary Material 3). The inverse association between tea consumption and CVD mortality was similar in men (ES, 0.84; 95% CI, 0.71 to 0.99) and women (ES, 0.86; 95% CI, 0.75 to 0.99). By geographic region, the largest reduction in CVD mortality was found in Asia (ES, 0.75; 95% CI, 0.65 to 0.88), but the difference was not statistically significant (p for all comparisons > 0.1) (Table 2). Some evidence for a non-linear association was found between tea consumption and CVD mortality (p for non-linearity<0.001) (Figure 5). Despite the significant non-linearity showing an identical degree of risk reduction for 1.5-3.0 cup/day, the ES decreased by 27% with up to 8.0 cups of tea consumption per day.

#### Cancer mortality

**Figure 6.**
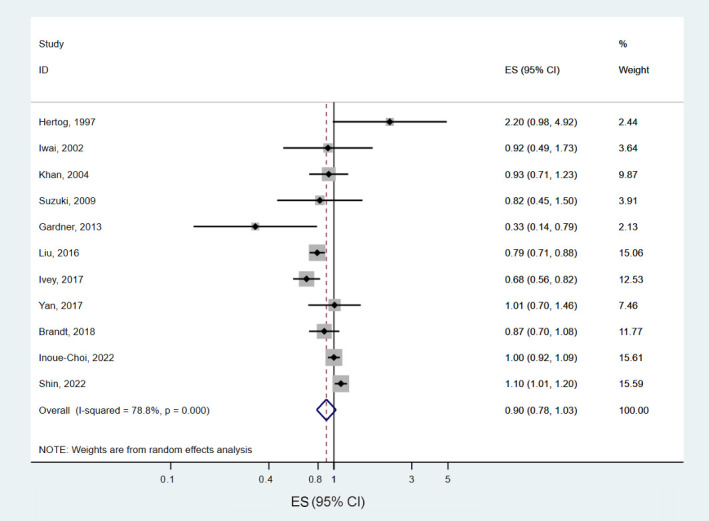
Forest plots of the prospective cohort studies of cancer mortality for high versus low tea consumption. ES, effect estimate; CI, confidence interval.

**Figure 7.**
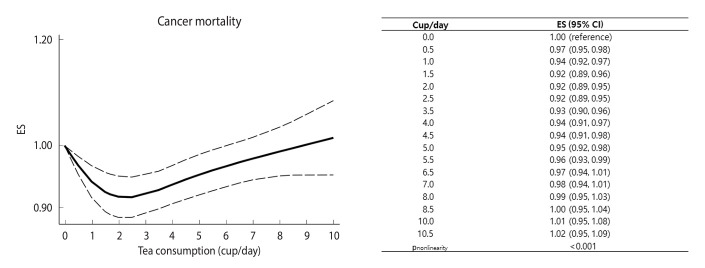
Pooled dose-response association between tea consumption and cancer mortality. Solid lines represent effect estimates (ESs); dashed lines represent 95% confidence intervals (CIs).

Twenty-two prospective cohort data sets (11 papers) involving 1,439,658 participants and 56,049 deaths examined the association between tea consumption and cancer mortality [9,13,14, 17,21-23,26,29,30]. The summary ES for cancer mortality, comparing the highest and lowest levels of tea consumption, was 0.90 (95% CI, 0.78 to 1.03), with significant heterogeneity among studies (*I*^2^ =78.8%, p<0.001) (Figure 6). When we calculated the pooled ES after excluding 1 study at a time, the ESs ranged from 0.87 (95% CI, 0.75 to 1.00) to 0.94 (95% CI, 0.82 to 1.07) (Supplementary Material 4). We found no significant difference by gender (p for difference=0.93) or geographic region (p for difference>0.2 in all comparisons) (Table 2). A significant non-linear association was found between tea consumption and cancer mortality (p for nonlinearity < 0.001) (Figure 7). The most robust inverse association was shown for tea consumption of 1.5-2.0 cup/day, and a significant reduction in cancer mortality was observed with tea consumption of up to 5.5 cup/day.

#### Publication bias

No evidence of publication bias was found for tea consumption and all-cause mortality (Begg’s p=0.97; Egger’s p=0.75), CVD mortality (Begg’s p>0.99; Egger’s p=0.65), or cancer mortality (Begg’s p=0.88; Egger’s p=0.43).

## DISCUSSION

The current systematic review and meta-analysis of 38 prospective cohort data sets (from 27 papers) with 1,956,549 total participants found inverse associations between tea consumption and all-cause and CVD mortality. People with the highest level of tea consumption had a 10% lower risk of all-cause mortality and a 14% lower risk of CVD mortality than those with the lowest consumption level. The results from the dose-response analyses showed a non-linear association between tea consumption and mortality from all causes, CVD, and cancer. The most significant reduction in all-cause mortality was observed at 2.0 cup/day of tea consumption, and further intake did not appear to further lower the risk of death. A plateau in CVD mortality was observed at moderate intake levels (1.5-3.0 cup/day of tea consumption), but a continued decline in mortality risk was observed at higher intake levels. For cancer mortality, the greatest reduction in mortality risk was observed at the level of consuming 1.5 cups of tea per day, and additional intake did not lower mortality.

Many previous observational studies have reported the association between tea drinking and the risk of chronic diseases. A recent meta-analysis of 19 prospective cohort studies, with a total of 1,076,300 participants, found that people consuming 4 or more cups of tea per day had a 17% lower risk of type 2 diabetes than people who did not drink tea [47]. For CVD risk, a meta-analysis of 7 prospective cohort studies involving 9,211 cases of coronary heart disease among 772,922 participants indicated that green tea consumers had less risk of coronary heart disease than those who did not consume green tea. The risk was 11% lower for 1 cup/day, 16% lower for 2 cup/day, 15% lower for 3 cup/day, and 12% lower for 4 cup/day [48]. Furthermore, a meta-analysis of 5 prospective cohort studies, including 11,421 stroke cases among 645,393 participants, reported that 900 mL/day of green tea consumption was associated with a 23% reduced risk of stroke [49]. Regarding risk of cancer, meta-analyses of prospective cohort studies showed that high tea consumption was inversely associated with several types of cancer, including glioma and ovarian, liver, bladder, and lymphoid cancer [50-52]. The lower risk of mortality with tea consumption observed in the current study may be due to the inverse association between tea consumption and the risks of many chronic diseases reported in previous studies.

A previous meta-analysis reported 26% lower and 24% lower risks of cardiac death and all-cause death, respectively, per 3-cup increment of tea consumption [34]. Another meta-analysis published in 2015 found that green tea consumption was inversely associated with mortality from CVD and all causes, and black tea consumption was inversely associated with mortality from cancer and all causes [33]. However, in that meta-analysis, when classifying the types of tea, the types of tea were estimated and analyzed according to the region where the study was conducted [33]. A recent meta-analysis from 2020 observed that each 1-cup increase in daily tea intake was associated with 4% and 2% lower risks of mortality from CVD and all causes, respectively [35]. Previous results and the results of our study are consistent in that tea consumption was inversely associated with the risk of CVD and all-cause mortality, and our findings indicated that consuming 1.5-2.0 cups of tea daily was associated with lower mortality rates from CVD, cancer, and all causes.

In the subgroup analysis by geographical region, the inverse association between tea consumption and risk of mortality from all causes was stronger in Asia than in Europe. These differences observed across regions may be due to regional differences in the types of tea that are widely consumed. Among the studies included in this meta-analysis, those that reported the type of tea were mostly from Asia, and it was primarily green tea. Although studies conducted in Europe did not report the type of tea, it is generally known that people in Europe and North America mainly consume black tea [1,53]. Green tea and black tea go through different manufacturing processes, and their composition varies accordingly. The most abundant polyphenols in green tea are catechins such as epicatechin, epicatechin-3-gallate, epigallocatechin, and epigallocatechin-3-gallate (EGCG). Meanwhile, the major polyphenolic components of black tea are theaflavin and thearubigin [53]. These differences in the composition of green tea and black tea may have influenced the difference in their associations with mortality risk. Most of the studies included in this meta-analysis did not report the ES by type of tea. The single study that reported ES by tea types found a significant inverse association between green tea consumption and mortality from all causes and CVD [27]. Specifically, drinking green tea 3 or more times a week was associated with a 21% lower risk of mortality from all causes and a 22% lower risk of mortality from CVD than drinking it fewer than 3 times a week. The researchers did not find a significant association between black tea consumption and mortality risk. It appears that more large-scale cohort studies need to be conducted in the future to determine whether mortality risk varies depending on the type of tea. Another explanation for the differences by geographical region in the association between tea consumption and mortality may be differences in how tea is consumed. In Europe, especially in the United Kingdom, it is common to add milk to tea [9]. Proteins in milk can combine with polyphenols in tea [54], reducing the polyphenols’ antioxidant capacity by inhibiting absorption and decreasing their bioavailability [55]. An experimental study found that the ferric iron-reducing antioxidant power value, an indicator of antioxidant activity, decreased as milk was added to tea [56]. The reduced antioxidant capacity of flavonoids after binding to proteins such as casein in milk may have attenuated the association between tea consumption and mortality. To understand more clearly the effects of adding milk to tea on the association between tea consumption and mortality, future prospective cohort studies that include information on how tea is consumed would need to be performed.

Because of the antioxidant properties of the polyphenols contained in tea, drinking tea has generally been considered a healthy dietary habit [57]. Antioxidative ability is an important factor in preventing disease and lowering the risk of premature death because oxidative stress is known to cause chronic disease by damaging normal cells and DNA. Tea flavonoids such as catechins or theaflavins have been found to decrease the oxidation of low-density lipoproteins, which cause atherosclerosis and increase the risk of CVD [58]. In addition, EGCG has been shown to have preventive effects against cancer by inhibiting the activity of urokinase, which invades cells and forms metastases in human cancers [59], or by inducing apoptosis and cell cycle arrest specific to cancer cells [60].

This study has offered a comprehensive meta-analysis of prospective cohort studies to examine the associations between tea consumption and the risks of mortality from all causes, CVD, and cancer. Prospective cohort studies have relatively less recall or selection bias than other observational study designs. The present meta-analysis included a large number of people (1,956,549 total participants), including recently published large cohorts, and thus, the results could have considerably more statistical power. To identify the appropriate tea consumption level for minimizing mortality risk, we performed a dose-response analysis that could show changes in ESs as tea intake increased. Additionally, unlike previous meta-analyses on the association between tea consumption and mortality, we excluded studies where all participants were patients suffering from a specific disease to examine the effect of tea consumption on mortality risk in the general population. Despite these strengths, several limitations should be mentioned as well. First, in most of the included studies, the data on tea consumption were assessed through a food frequency questionnaire, which allowed the possibility of misclassification. Fortunately, such misclassification of dietary intake tends to lead to a null result, so it is unlikely that the associations between tea consumption and mortality risk were exaggerated. Second, the volume of tea consumption was usually reported in cups, but cup size may differ from study to study. Third, the chemical composition of each cup of tea, such as its polyphenols, could vary by season, climate, and plant age, which may affect the associations between tea consumption and the risk of mortality [53]. Finally, the adjustment factors differed across the studies, which might have affected the pooled estimates in the meta-analysis. Most included studies controlled for potential confounders including smoking, alcohol consumption, and BMI, but the possibility of additional confounding factors cannot be completely ruled out.

## CONCLUSION

The current systematic review and meta-analysis of prospective cohort studies suggested that moderate daily tea consumption (1.5-2.0 cup/day) was associated with lower risks of mortality from all causes, CVD, and cancer in the general population. Future well-designed, large-scale prospective cohort studies analyzing different types of tea are needed to develop dietary guidelines for tea consumption to reduce the risk of chronic disease and premature death.

## Figures and Tables

**Figure f8-epih-46-e2024056:**
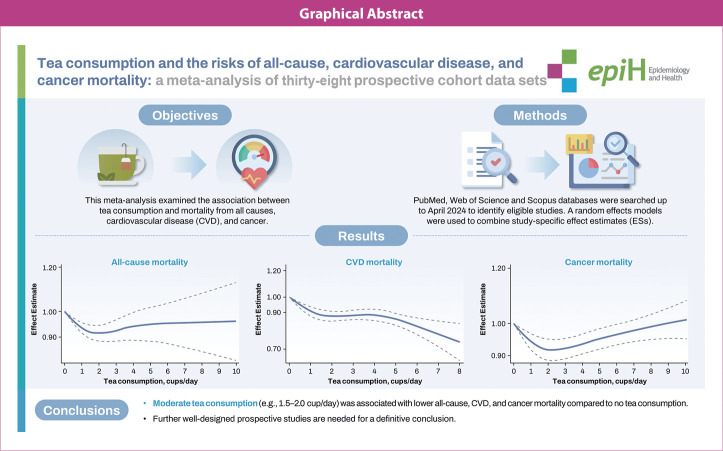


**Table 1.** Characteristics of studies included in the meta-analysis of tea consumption and mortality from all causes, CVD, and cancer

Study	Country	Cohort name	Follow-up period (yr)	Age at baseline (yr)	Study size		Gender	Tea consumption	Cause of death	Adjustment for covariates
					Subjects	No. of deaths				
Kahn et al., 1984 [6]	USA	Seventh-Day Adventists	21	≥30	21,022	5,679	Men and women	<1 vs. ≥1 cup/day	All causes	Age, gender, smoking history, history of heart disease, stroke, hypertension, diabetes, or cancer; age at initial exposure to the Adventist Church
Hertog et al., 1993 [7]	Netherlands	Zutphen Elderly Study	5	65-84	805	43	Men	0-250 vs. >500 mL/day	CHD	Age, intake of total energy, saturated fatty acids, cholesterol, alcohol, coffee, vitamin C, vitamin E, beta-carotene, dietary fiber, history of myocardial infarction, BMI, smoking, serum total and HDL cholesterol, systolic blood pressure
Klatsky et al., 1993 [8]	USA	Northern California Kaiser Permanente Medical Care Program	8	N/A	125,356	4,208	Men and women	0 vs. ≥4 cup/day	All causes	Age, gender, BMI, smoking, alcohol, race, education, marital status
Hertog et al., 1997 [9]	UK	Caerphilly study	14	45-59	1,900	338^3^	Men	0-300 vs. >1,200 mL/day	All causes	Age, smoking, social class, BMI, intakes of total energy, alcohol, fat, vitamin C, vitamin E, and β-carotene
						104^4^			Cancer	
Woodward et al., 1999 [10]	Scotland	Scottish Heart Health Study	7.7	40-59	11,507	588^3^	Men and women	0 vs. ≥5 cup/day	All causes	Age, housing tenure, activity at work, activity in leisure, cigarette smoking status, BMI, Bortner score, cotinine, systolic blood pressure, fibrinogen, total cholesterol, HDL cholesterol, triglycerides, alcohol, vitamin C, and coffee
						206^5^			CHD	
Nakachi et al., 2000 [11]	Japan	Saitama	11	>40	8,552	222	Men and women	≤3 vs. ≥10 cup/day	CVD	Age, cigarette smoking, alcohol consumption, intake of meat, and relative body weight
		Prefecture								
Hirvonen et al., 2001 [12]	Finland	Alpha-Tocopherol, Beta-Carotene Cancer Prevention	6.1	50-69	25,372	815	Men	<1 vs. ≥1 cup/day	CVD	Age, supplementation group, systolic and diastolic blood pressure, serum total cholesterol, serum HDL cholesterol, BMI, smoking years, number of cigarettes smoked daily, history of diabetes mellitus and CHD, marital status, education, leisure-time physical activity
		Study								
Iwai et al., 2002 [13]	Japan	Tottori Prefecture	9.9	40-79	2,855	361^3^	Men and women	<0.5 vs. ≥4 cup/day	All causes	Age, smoking, alcohol, history of selected diseases, physical activity, educational status
						61^4^			Cancer	
Khan et al., 2004 [14]	Japan	Hokkaido	13.8	≥40	3,158	244	Men and women	Never+several times/yr+several times/mo vs. several times/wk+daily	Cancer	Age, health education, health examination, health status, smoking
		Prefecture								
Andersen et al., 2006 [15]	USA	Iowa Woman’s Health Study	15	55-69	27,312	4,265^3^	Women	0 vs. >3 cup/day	All causes	Age, smoking, intake of alcohol, BMI, waist-hip ratio, education, physical activity, use of estrogens, use of multivitamin supplements, energy intake, and intakes of whole and refined grain, red meat, fish, seafood, total fruit and vegetables
						1,411^6^			CVD	
Paganini-Hill et al., 2007 [16]	USA	Leisure World Cohort Study	23	≥44	13,624	11,386	Men and women	0 vs. ≥2 cup/day	All causes	Age, gender, smoking, exercise, BMI, alcohol intake, and histories of hypertension, angina, heart attack, stroke, diabetes, rheumatoid arthritis, and cancer
Suzuki et al., 2009 [17]	Japan	Shizuoka Elderly Cohort	6	65-84	12,251	1,224^3^	Men and women	<1 vs. ≥7 cup/day	All causes	Age, gender, smoking status, alcohol consumption, BMI, frequency of physical activity
						405^6^			CVD	
						400^4^			Cancer	
de Koning Gans et al., 2010 [18]	Netherlands	European Prospective Investigation into Cancer and Nutrition-Netherlands (EPIC-NL), MORGEN	13	20-69	37,514	1,405^3^	Men and women	<1 vs. >6 cup/day	All causes	Age, gender, cohort (strata), education, physical activity, smoking status, waist circumference, menopausal status, alcohol, coffee, vitamin C, level, fiber, consumption, energy, saturated fat
						123^6^			CVD	
Mineharu et al., 2011 [19]	Japan	Japan Collaborative Cohort Study for Evaluation of Cancer Risk (JACC)	13.1	40-79	82,655	3,125	Men and women	<1 cup/wk vs. ≥6 cup/day	CVD	Age, BMI, smoking status, alcohol intake, history of hypertension, history of diabetes, education, waking hours, hours of sports participation, perceived mental stress, multivitamin use, vitamin E supplement use; total consumption of fruits, vegetables, beans, meat, and fish; and total daily energy intake
Bertoia et al., 2013 [20]	USA	Women’s Health Initiative Observational Study (WHI)	11	50–79	92,847	205	Women	0 vs. ≥4 cup/day	CVD	Age, total energy intake, race, income, smoking status, physical activity, waist-to-hip ratio, BMI, atrial fibrillation, coronary artery disease, heart failure, diabetes, high cholesterol, hypertension, pulse in 60 seconds, and hormone use
Gardner et al., 2013 [21]	USA	Northern Manhattan Study	11	>40	2,461	863^3^	Men and women	1 cup/mo vs. ≥2 cup/day	All causes	Age, gender, BMI, race, education, pack-years of smoking, alcohol consumption, energy, protein, carbohydrates, total fat, saturated fat, history of vascular risk factors, other non-water beverage consumption, coffee additives (milk, cream, nondairy creamer), coffee
						342^6^			CVD	
						160^4^			Cancer	
Liu et al., 2016 [22]	China	Chinese Prospective Smoking Study	11	>40	164,681	32,700	Men	0 vs. >10 g/day	All causes	Age, BMI, marital status, urban locality, education, job status, smoking status, alcohol drinking; times of weekly consumption for fish, meat, poultry consumption, egg, and milk; black tea drinker, jasmine tea drinker, other tea drinker
									CVD	
									Cancer	
Ivey et al., 2017 [23]	USA	Nurses’ Health Study II	18	25-42	93,145	1,894^3^	Women	0 vs. >1 time/wk	All causes	Age, BMI, smoking status, menopausal status, family history (of diabetes, cancer, and myocardial infarction), multivitamin supplement use, aspirin use, race, type 2 diabetes, hypercholesterolemia, hypertension, physical activity, energy intake, alcohol consumption, and the Alternative Health Eating Index (minus alcohol) score
						189^6^			CVD	
						887^4^			Cancer	
Lim et al., 2017 [24]	Australia	Calcium Intake Fracture Outcome Study	10	≥70	1,055	362^3^	Women	1 cup/day increment	All causes	Age, smoking history, SES, diabetes status, hypertension, blood pressure, prevalent CVD, medications and treatment code (calcium supplementation vs. no calcium supplementation), fluid status, BMI, estimated glomerular filtration rate
						142^6^			CVD	
Yan et al., 2017 [25]	USA	Aerobics Center Longitudinal Study	16	20-82	11,808	842^3^	Men and women	0 vs. >14 cup/wk	All causes	Age, gender, baseline examination year, regular coffee use, decaffeinated coffee use, herbal tea use, physical inactivity, BMI, smoking, alcohol consumption, metabolic equivalents
						250^6^			CVD	
						345^4^			Cancer	
van den Brandt, 2018 [26]	Netherlands	Netherlands Cohort Study (NLCS)	10	55-69	120,852	8,665^3^	Men and women	0 vs. ≥5 cup/day	All causes	Age, cigarette smoking status, number of cigarettes smoked per day, years of smoking, history of physician-diagnosed hypertension and diabetes, body height, BMI, non-occupational physical activity, highest level of education, intake of alcohol, nuts, vegetables and fruit, tea, energy, use of nutritional supplements, postmenopausal hormone replacement therapy (in women)
						2,927^6^			CVD	
						3,849^4^			Cancer	
Wang et al., 2020 [27]	China	China-PAR project (3 cohorts)^1^	7.3	52, mean	100,902	5,479^3^	Men and women	<3 vs. ≥3 time/wk	All causes	Age, gender, region, residential area, cohort, educational level, family history of atherosclerotic CVD, smoking, drinking, physical activity level, dietary factors, BMI, systolic blood pressure, fasting blood glucose, total cholesterol, HDL cholesterol
						1,477^6^			CVD	
Teramoto et al., 2021 [32]	Japan	Japan Collaborative Cohort Study for Evaluation of Cancer Risk (JACC)	18.5	40-79	44,521	8,666	Men and women	0 vs. ≥7 cup/day	All causes	Age, gender, coffee consumption, history of hypertension, history of diabetes, BMI, smoking status, alcohol consumption, hours of exercise, hours of walking, perceived mental stress, educational level, regular employment, dietary intakes of vegetable, fish, fruits, and soybeans
Chen et al., 2022 [28]	UK	UK Biobank	12.1	37-73	498,158	34,699^3^	Men and women	0 vs. ≥5 cup/day	All causes	Age, gender, ethnicity, educational level, BMI, smoking status, alcohol intake frequency, physical activity, dietary pattern, general health status, hypertension, diabetes, depression, coffee consumption
						6,663^6^			CVD	
Inoue-Choi et al., 2022 [29]	UK	UK Biobank	11.2	40-69	498,043	15,790	Men and women	0 vs. ≥10 cup/day	Cancer	Age, gender, race and ethnicity, Townsend deprivation score, general health status, cancer, CVD, diabetes, BMI, tobacco smoking, physical activity, alcohol intake, coffee intake; dietary intake including vegetables, fruits, red meat, and processed meat; assessment centers
Shin et al., 2022 [30]	Asia	Asia Cohort Consortium (12 cohorts)^2^	6.5-22.7	54.3 mean	528,504	94,744^3^	Men and women	0 vs. ≥5 cup/day	All causes	Age, BMI, smoking status, alcohol intake, educational level, energy intake, coffee consumption
						22,850^6^			CVD	
						27,207^4^			Cancer	
Qiu et al., 2023 [31]	China	China Health and Nutrition Survey (CHNS)	17.9	54.4 mean	6,387	580	Men and women	0 vs. 3-4 cup/day	All causes	Age, gender, marital status, educational level, nationality, residential area, smoking status, occupation, level of annual income, mean systolic blood pressure, mean BMI, mean waist and hip circumferences, hypertension, diabetes mellitus, myocardial infarction, stroke, malignant tumor

CVD, cardiovascular disease; CHD, coronary heart disease; BMI, body mass index; HDL, high-density lipoprotein; SES, socioeconomic status; N/A, not available.

1 China 3 cohort is China Multi-Center Collaborative Study of Cardiovascular Epidemiology, International Collaborative Study of CVD in Asia, and Community Intervention of Metabolic Syndrome in China & Chinese Family Health Study.

2 Asia Cohort Consortium includes Japan Public Health Center-based Prospective Study (JPHC) I, II, Miyagi Cohort, Ohsaki National Health Insurance Cohort Study, Life Span Study Cohort (RERF), 3 Prefecture Miyagi, 3 Prefecture Aichi, Seoul Male Cancer Cohort (Seoul Male), Korean Multi-center Cancer Cohort Study (KMCC), Shanghai Men’s Health Study (SMHS), Shanghai Women’s Health Study (SWHS), and Singapore Chinese Health Study (SCHS).

3 Death from all causes.

4 Death from cancer.

5 Death from CHD.

6 Death from CVD.

**Table 2.** Summary of pooled ESs of mortality from all causes, CVD, and cancer by tea consumption

Variables	No. of studies	ES (95% CI)	p for difference
All-cause mortality			
High vs. low tea consumption			
All studies	20	0.90 (0.86, 0.95)	
Stratified by gender			
Men	8	0.87 (0.77, 0.98)	0.97
Women	9	0.90 (0.79, 1.01)	
Stratified by geographical region			
Asia	7	0.84 (0.77, 0.91)	
USA	7	0.92 (0.83, 1.02)	0.32^1^
Europe	5	1.12 (0.88, 1.42)	0.05^1^
Oceania	1	0.92 (0.86, 0.98)	0.57^1^
Stratified by follow-up years			
≥Median	10	0.96 (0.88, 1.05)	0.17
<Median	10	0.86 (0.81, 0.92)	
Stratified by no. of subjects			
≥Median	10	0.89 (0.85, 0.94)	0.87
<Median	10	0.91 (0.80, 1.04)	
CVD mortality			
High vs. low tea consumption			
All studies	18	0.86 (0.79, 0.94)	
Stratified by gender			
Men	10	0.84 (0.71, 0.99)	0.83
Women	11	0.86 (0.75, 0.99)	
Stratified by geographical region			
Asia	6	0.75 (0.65, 0.88)	
USA	5	0.91 (0.72, 1.14)	0.31^2^
Europe	6	1.00 (0.78, 1.26)	0.17^2^
Oceania	1	0.90 (0.79, 0.94)	0.55^2^
Stratified by follow-up years			
≥Median	11	0.86 (0.80, 0.92)	0.76
<Median	7	0.84 (0.68, 1.03)	
Stratified by no. of subjects			
≥Median	9	0.84 (0.79, 0.89)	0.76
<Median	9	0.84 (0.69, 1.03)	
Cancer mortality			
High vs. low tea consumption			
All studies	11	0.90 (0.78, 1.03)	
Stratified by gender			
Men	7	0.91 (0.77, 1.09)	0.93
Women	5	0.93 (0.70, 1.23)	
Stratified by geographical region			
Asia	5	0.92 (0.74, 1.15)	
USA	3	0.70 (0.46, 1.07)	0.25^3^
Europe	3	1.00 (0.80, 1.24)	0.64^3^
Stratified by follow-up years			
≥Median	6	0.97 (0.82, 1.14)	0.24
<Median	5	0.80 (0.70, 0.92)	
Stratified by no. of subjects			
≥Median	6	0.88 (0.76, 1.03)	0.72
<Median	5	0.94 (0.65, 1.35)	

ES, effect estimate; CVD, cardiovascular disease; CI, confidence interval.

1 ESs of all-cause mortality for USA vs. Asia (p=0.32), Europe vs. Asia (p=0.05), and Oceania vs. Asia (p=0.57).

2 ESs of CVD mortality for USA vs. Asia (p=0.31), Europe vs. Asia (p=0.17), and Oceania vs. Asia (p=0.55).

3 ESs of cancer mortality for USA vs. Asia (p=0.25) and Europe vs. Asia (p=0.64).

## Supplementary materials

Supplementary Material 1. Quality assessment scores according to Newcastle-Ottawa Quality Assessment Scale.

Supplementary Material 2. Sensitivity analysis results by excluding one study at a time for the association between highest versus lowest levels of tea consumption and all-cause mortality.

Supplementary Material 3. Sensitivity analysis results by excluding one study at a time for the association between highest versus lowest levels of tea consumption and CVD mortality.

Supplementary Material 4. Sensitivity analysis results by excluding one study at a time for the association between highest versus lowest levels of tea consumption and cancer mortality.
