# Supplementary material for: Tea consumption and risk of all-cause, cardiovascular disease, and cancer mortality: a meta-analysis of thirty-eight prospective cohort data sets
Source: Epidemiol Health. 2024 Jun 21;46:e2024056. doi: 10.4178/epih.e2024056 (PMC11573487; doi:10.4178/epih.e2024056)
Supplement: Supplementary Material 1. — Quality assessment scores according to Newcastle-Ottawa Quality Assessment Scale. [file epih-46-e2024056-Supplementary-1.docx]

**Supplementary Material 1.** Quality assessment scores according to Newcastle-Ottawa Quality Assessment Scale

| Criteria | **Selection of subjects** | **Comparability of cohorts** | **Ascertainments of outcomes of interests** | **Total** |
| --- | --- | --- | --- | --- |
| Kahn, 1984 | 3 | 2 | 2 | 7 |
| Hertog, 1993 | 4 | 2 | 3 | 9 |
| Klatsky, 1993 | 3 | 2 | 3 | 8 |
| Hertog, 1997 | 3 | 2 | 3 | 8 |
| Woodward, 1999 | 3 | 2 | 3 | 8 |
| Nakachi, 2000 | 3 | 2 | 2 | 7 |
| Hirvonen, 2001 | 2 | 2 | 3 | 7 |
| Iwai, 2002 | 3 | 2 | 3 | 8 |
| Khan, 2004 | 3 | 2 | 3 | 8 |
| Andersen, 2006 | 3 | 2 | 3 | 8 |
| Paganini-Hill, 2007 | 3 | 2 | 3 | 8 |
| Suzuki, 2009 | 3 | 2 | 2 | 7 |
| Koning Gans, 2010 | 3 | 2 | 3 | 8 |
| Mineharu, 2011 | 3 | 2 | 2 | 7 |
| Gardner, 2013 | 3 | 2 | 2 | 7 |
| Bertoia, 2013 | 3 | 2 | 2 | 7 |
| Liu, 2016 | 3 | 2 | 3 | 8 |
| Ivey, 2017 | 2 | 2 | 3 | 7 |
| Lim, 2017 | 3 | 2 | 3 | 8 |
| Yan, 2017 | 3 | 2 | 3 | 8 |
| Brandt, 2018 | 3 | 2 | 3 | 8 |
| Wang, 2020 | 4 | 2 | 3 | 9 |
| Teramoto, 2021 | 3 | 2 | 2 | 7 |
| Chen, 2022 | 3 | 2 | 2 | 7 |
| Inoue-Choi, 2022 | 3 | 2 | 2 | 7 |
| Shin, 2022 | 3 | 2 | 2 | 7 |
| Qiu, 2023 | 4 | 2 | 3 | 9 |
