# Supplementary material for: Tea consumption and risk of all-cause, cardiovascular disease, and cancer mortality: a meta-analysis of thirty-eight prospective cohort data sets
Source: Epidemiol Health. 2024 Jun 21;46:e2024056. doi: 10.4178/epih.e2024056 (PMC11573487; doi:10.4178/epih.e2024056)
Supplement: Supplementary Material 4. — Sensitivity analysis results by excluding one study at a time for the association between highest versus lowest levels of tea consumption and cancer mortality. [file epih-46-e2024056-Supplementary-4.docx]

**Supplementary Material 4.** Sensitivity analysis results by excluding one study at a time for the association between highest *versus* lowest levels of tea consumption and cancer mortality.
